# First record of the genus *Discorhabdella* (Porifera, Demospongiae, Poecilosclerida, Crambeidae) from Sagami Bay, Japan with description of two new species

**DOI:** 10.3897/zookeys.1076.37278

**Published:** 2021-12-09

**Authors:** Yuji Ise, Jean Vacelet, Takato Izumi, Sau Pinn Woo, Shau Hwai Tan

**Affiliations:** 1 Centre for Marine & Coastal Studies (CEMACS), Universiti Sains Malaysia, 11800 USM, Penang, Malaysia Universiti Sains Malaysia Penang Malaysia; 2 Institut Méditerranéen de Biodiversité et d’Ecologie Marine et Continentale, CNRS, Aix Marseille Université, IRD, Avignon Université, Station Marine d’Endoume, Marseille, France Aix Marseille Université Marseille France; 3 Department of Biological Sciences, The University of Tokyo, Bunkyo-ku, Tokyo 113-0033, Japan The University of Tokyo Tokyo Japan

**Keywords:** biodiversity, Central Kuroshio Current, northwest Pacific, relict species, sponge taxonomy, Tethys Sea

## Abstract

Two new species of *Discorhabdella* are described from Sagami Bay, Japan. *Discorhabdella* has been suggested to have an ancient Tethyan origin according to discovery of their unique pseudoastrose acanthostyles from late Eocene to Oligocene deposits. This is the first record of the genus from the northwest Pacific and first record of the family Crambeidae from Japan. *Discorhabdellahispida***sp. nov.** is distinctive within the genus by possession of special sigmoid microscleres and C-shaped isochelae with short alae. *Discorhabdellamisakiensis***sp. nov.** is characterized by short choanosomal subtylostyles, and their length overlapped with that of the ectosomal subtylostyles. Only one other species, *Discorhabdellatuberosocapitata* (Topsent, 1890), has the same spicule composition. However, all spicule types are larger in *D.tuberosocapitata* than those of *D.misakiensis***sp. nov.**, and the shape of the isochelae is different: the alae are more widely opened in *D.tuberosocapitata*. An identification key to species of the genus *Discorhabdella* is also provided. The discovery of two new species from warm temperate northwest Pacific extends the geographical distribution of the genus *Discorhabdella*.

## Introduction

Sponges of the genus *Discorhabdella* Dendy, 1924 are characterized by the possession of smooth ectosomal subtylostyles, long choanosomal styles/subtylostyles with swollen lumpy bases, and tuberculate club-shaped pseudoastrose or heavily spined acanthostyles that form an erect hymedesmioid skeleton and various cheloid microscleres (Maldonado et al. 2001, Van Soest 2002). It has been suggested that *Discorhabdella* originated in the Tethys Sea (Boury-Esnault et al. 1992, Maldonado and Uriz 1996, Maldonado et al. 2001), as their unique pseudoastrose acanthostyles were discovered from the late Eocene to Oligocene deposits in New Zealand (Hinde and Holmes 1892, Łukowiak 2015, 2016). Seven extant species are currently known from the genus (Van Soest et al. 2019). Of these, *Discorhabdellaincrustans* Dendy, 1924 is reported only from its type locality Three King’s Islands, New Zealand. *Discorhabdellalittoralis* Maldonado, Carmona, Van Soest & Pomponi, 2001 and *D.urizae* Maldonado, Carmona, Van Soest & Pomponi, 2001 are reported from off the Pacific coast of Panama. *Discorhabdellaurizae* is also reported from Gulf of California (Aguilar-Camacho and Carballo 2012). *Discorhabdellahindei* Boury-Esnault, Pansini & Uriz, 1992 is reported from the Alboran Sea, and *D.tuberosocapitata* (Topsent, 1890) is reported from Azores, Canaries and Madeira (Van Soest 2002, Van Soest et al. 2019). Two recently described species have been discovered from north of Madagascar (*D.pseudaster* Vacelet & Cárdenas, 2018) and Gulf of Mexico (*D.ruetzleri* Díaz & Pomponi, 2018). A recent faunal survey of benthic animals in Sagami Bay, Japan yielded several undescribed species of the family Crambeidae (Ise 2017), and the descriptions of two new species of *Discorhabdella* are provided herein.

## Materials and methods

**Figure 1.**
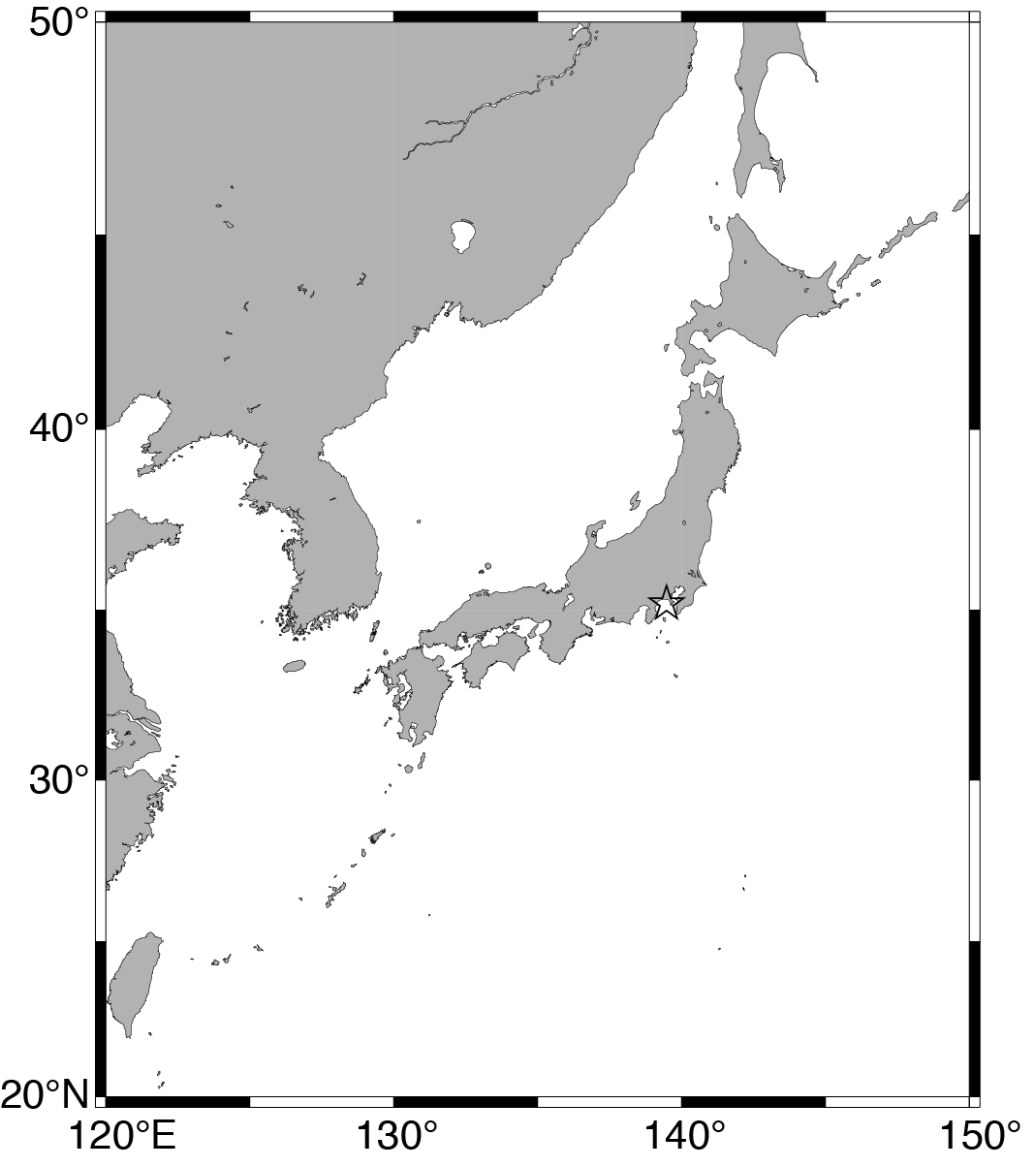
Location of sampling site. Open star indicates Sagami Bay.

The sponges described in the present study were collected by dredging from the R/V Rinkai-maru of Misaki Marine Biological Station, the University of Tokyo. The sampling was carried out at the northeastern part of Sagami Bay during the period of 10–13 January 2012 (Fig. 1). The specimens were kept alive in seawater for several hours and directly preserved in 90% ethanol afterwards. Dry fragments of the sponge were digested using hydrogen peroxide in order to obtain clean spicules. They were then cleaned using distilled water, centrifuged, and resuspended three times. Cleaned spicules were then placed on glass slides, dried, embedded in mounting medium Eukitt® (O. Kindler), cover-slipped, and then observed under a light microscope. Spicules were also placed on copper stub, coated with 400Å platinum, and observed by scanning electron microscope (JEOL JSM-6380LV). Spicules were measured with calibrated ocular micrometer directly under a microscope. Measurements were carried out along randomly chosen transects across the slide, ignoring unfocused, broken, or malformed spicules. Measurements of choanosomal subtylostyles of *Discorhabdellahispida* sp. nov. were only taken from the width of base and shaft as they are usually broken during spicule preparation steps. About 30 spicules for each type of spicule were measured. Spicule sizes are given as a range, followed by the mean in parenthesis. Spicule and morphological nomenclature follows Boury-Esnault and Rützler (1997), and terminology of cheloid microscleres follows Hajdu et al. (1994). Terminology for geographical distribution of each species basically follows descriptions of the original references; however, the data are corrected in Table 1 according to Marine Ecoregions of the World (Spalding et al. 2007). Specimens were deposited in National Museum of Nature and Science, Tsukuba, Japan (NSMT).

**Table 1.** Morphological comparison of spicules and geographical distribution of extant *Discorhabdella* species. Locality is described as ecoregions and province following Spalding et al. (2007). Spicule sizes are given as the range, followed by the mean in parenthesis. All spicule measurements in µm.

**Species**	**Locality**	**Depth (m)**	**Ectosomal subtylostyle**	**Choanosomal style/subtylostyle**	**Pseudoastrose acanthostyle or acanthostyle**	**Isochelae**	**Sigma**	**spined microxea**	**Other microsclere**	**Reference**
			**length × width**	**length × width**	**length × base width**	**length, number of alae**	**length × width**	**length × width**	**length**	
*D.hindei* Boury-Esnault et al., 1992	Alboran Sea, Mediterranean Sea	534–604	276–445 (367) × 5.2	855–1556 (1086) × 34–52 (43)	43–57 (48) × 36–39 (38.5)	22–27 (23), 8 alae	11–16 (12) × 1–1.3 (1.3)	none	none	Boury-Esnault et al. 1992
*D.incrustans* Dendy, 1924	Three King‘s North Cape, Northern New Zealand	180	357–592 (496.8) × 10–15 (12.6)	900–1700 × 28–61 (43.1)	36–53 (45.4) × 32–43 (37.2)	33–51 (44), up to 7 alae	none	26–34 (31.4)	none	Van Soest 2002
*D.littoralis* Maldonado et al., 2001	Nicoya, Tropical East Pacific	10–30	130–180 × 2.5–4	117–300 × 5–10	26–40 × 10–18.5	none	13–15 × 1	none	none	Maldonado et al. 2001
*D.pseudaster* Vacelet & Cárdenas, 2018	Western and Northern Madagascar, Western Indian Ocean	346–349	240–370, 9–10	more than 600 × 40–56	35–45 × 35–45	12–15, 4–5 alae	none	none	pseudoaster, 12.5–18 in diameter	Vacelet and Cárdenas 2018
*D.ruetzleri* Díaz & Pomponi, 2018	Floridian, Tropical Northwestern Atlantic	60–80	260–340 (300) × 3–7.55 (4)	470–810 (598) × 5–13 (10.5)	17–40 (29.6) × 7.5–20 (15)	20–25, unknown	12–18	15–18	none	Díaz and Pomponi 2018
*D.tuberosocapitata* (Topsent, 1890)	Azores Canaries Madeira, Lusitanian	550–736	330	c.a. 650 × c.a. 28	c.a. 130	25, 4–8 alae	none	none	none	Boury-Esnault et al. 1992, Van Soest 2002
*D.urizae* Maldonado et al., 2001	Nicoya, Tropical East Pacific	55–73	180–220 × 5–7	380–750 × 19–42	23–37 × 15–26	26–29, 5 alae	13–16 × 1	19–26 × 2–3	none	Maldonado et al. 2001
	Cortezian, Warm Temperate Northeast Pacific	344	175–220 (197.5) × 2.5–7.5 (4.75)	220–610 (423.3) × 17.5–35 (25.8)	30–42.5 (36.6) × 23–37	35–42 (36.6), 3 alae	15–20 (17.1)	15–22.5 (21.6)	none	Aguilar-Camacho and Carballo 2012
*D.hispida* **sp. nov.**	Central Kuroshio Current, Warm Temperate Northwest Pacific	113–223	292.2–392.5 (335.4) × 13.4–16.7 (15.2)	814–1500 × 42.0–56.5 (50.3)	84–127.5 (103.6) × 41.1–57.7 (48.0)	27.3–38 (31.7), 3–7 alae	none	none	sigmoid microsclere 20.7–31.2 (26.3)	this study
*D.misakiensis* **sp. nov.**	Central Kuroshio Current, Warm Temperate Northwest Pacific	255–318	203–257 (232) × 10.6–14.1 (11.7)	252.0–336.4 (295.2) × 18.6–26.6 (22.6)	73.0–91.3 (82.0) × 27.9–42.0 (34.2)	17.5–21.9 (19.8), 6 alae	none	none	none	this study

## Results

### Phylum Porifera Grant, 1836

**Class Demospongiae Sollas, 1885**

**Order Poecilosclerida Topsent, 1928**

**Family Crambeidae Lévi, 1963**

#### Genus Discorhabdella

Taxon classificationAnimaliaPoeciloscleridaCrambeidae

Dendy, 1924

6ADC38FB-8B60-50CB-968A-E5EF3049DFAF

##### Diagnosis.

Smooth ectosomal subtylostyles, long choanosomal styles/subtylostyles with swollen lumpy bases and tuberculate club-shaped pseudoastrose or heavily spined acanthostyles forming erect hymedesmioid skeleton; microscleres anchorate unguiferous isochelae and may include spined microxea with two lumpy swellings or sigma-like spicules (slightly modified from Van Soest 2002).

##### Type species.

*Discorhabdellaincrustans* Dendy, 1924: 376 (by monotypy).

#### Discorhabdella hispida sp. nov.

Taxon classificationAnimaliaPoeciloscleridaCrambeidae

78C4541D-E956-51EC-8FD0-8E31E59E6FDE

http://zoobank.org/025E3E24-8A78-4AD1-9BD6-44FD92B55A35

Figs 2A–C , 3 , 4

##### Material examined.

***Holotype*.**NSMT-Po-2489. Off Misaki, eastern part of Sagami Bay (Fig. 1), Japan (35°7.484'N, 139°33.212'E to 35°7.504'N, 139°33.625'E), 223–113 m depth, dredge, 13 January 2012.

##### Description of holotype.

**Figure 2.**
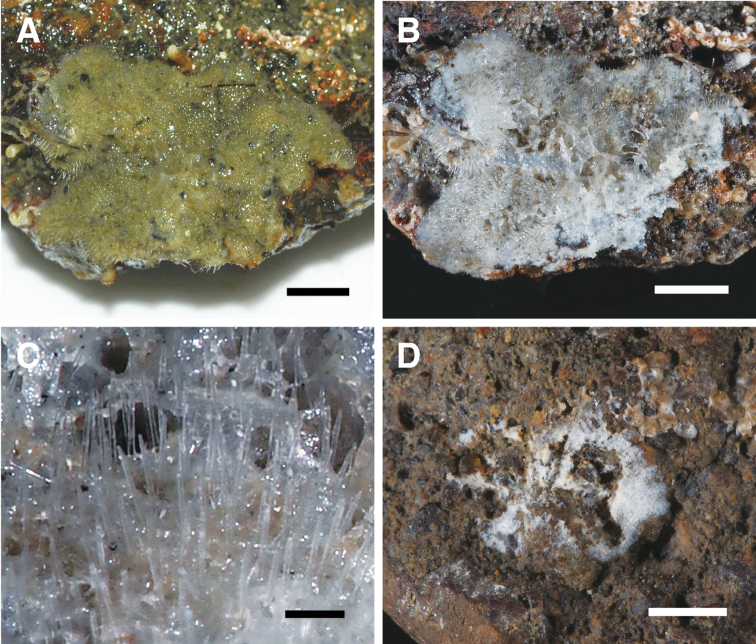
**A–C** External view of *Discorhabdellahispida* sp. nov., holotype (NSMT-Po-2489). **A** Alive **B** in ethanol preserved **C** magnified view of surface of preserved specimen. Note a number of choanosomal subtylostyles vertically protruding with their tips outward **D** external view of *Discorhabdellamisakiensis* sp. nov., holotype (NSMT-Po-2490) in ethanol preserved state. Note most part of the sponge was already used for spicule preparation. Scale bars: 5 mm (**A, B**); 500 µm (**C**); 3 mm (**D**).

***External morphology*.** Thinly encrusting, surface hispid due to protruding choanosomal large subtylostyles. Color greenish ochre in life, grayish white in ethanol. Size, 22 × 17 mm, about 0.3 mm thick (Fig. 2A–C). Oscules not observed in the living specimen; probably contracted in preserved state. Ostia observed only in preserved specimen, rounded, evenly distributed, 150–300 µm in diameter.

***Skeleton*.** Hymedesmioid skeleton made by large choanosomal subtylostyles making the sponge surface hispid and by perpendicular acanthostyles with their bases attached on substrate. Ectosomal subtylostyles arranged perpendicular to surface with tips outward. Anchorate unguiferous isochelae and sigmoid microscleres roughly dispersed throughout the sponge.

**Figure 3.**
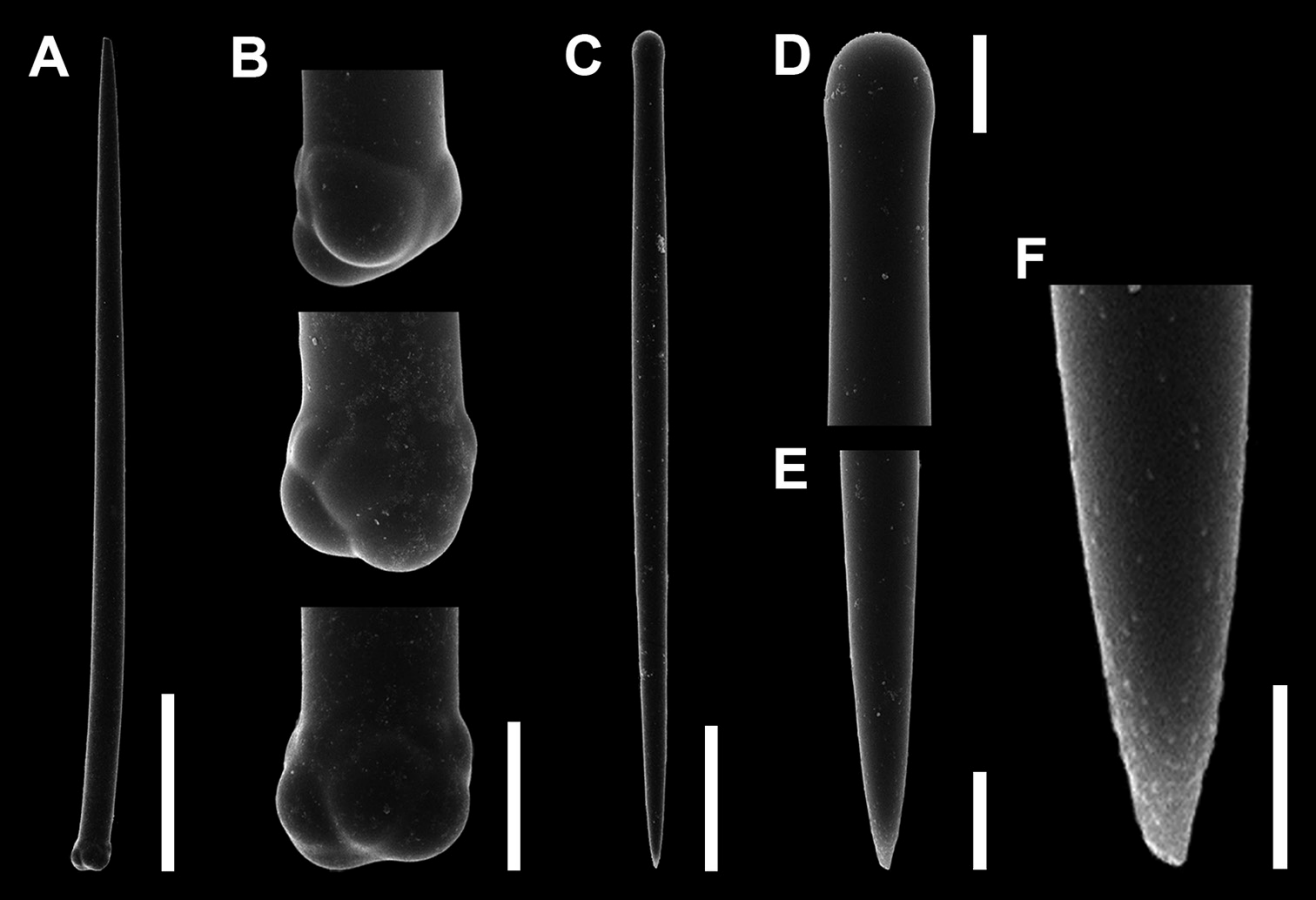
Spicules of *Discorhabdellahispida* sp. nov., holotype (NSMT-Po-2489). **A** Choanosomal subtylostyle **B** magnified view of base of subtylostyle **C–E** ectosomal subtylostyle **D** tyle **E** tip **F** magnified view of tip. Note the surface is microspined. Scale bars: 300 µm (**A**); 50 µm (**B**); 10 µm (**D, E**); 5 µm (**F**).

***Spicules*.** Choanosomal subtylotyles (Fig. 3A, B), long slightly curved near the base, maximum diameter at the base gradually tapering to sharp point (Fig. 3A). Base smooth and slightly lumpy (Fig. 3B). Size, 814–1500 µm in length, 42.0–56.5 (50.3) µm in shaft width, 52.4–70.8 (61.7) µm in base width.

Ectosomal subtylostyles (Fig. 3C–F), fusiform, smooth and straight; with smooth and slightly swollen base (Fig. 3D). Maximum diameter at middle region, then gradually tapering to a sharp point (Fig. 3E). Microspined sparsely around the shaft and densely around the tip (Fig. 3F). Size, 292.2–392.5 (335.4) µm in length, 13.4–16.7 (15.2) µm in shaft width, 10.7–14.0 (12.9) in tyle width.

**Figure 4.**
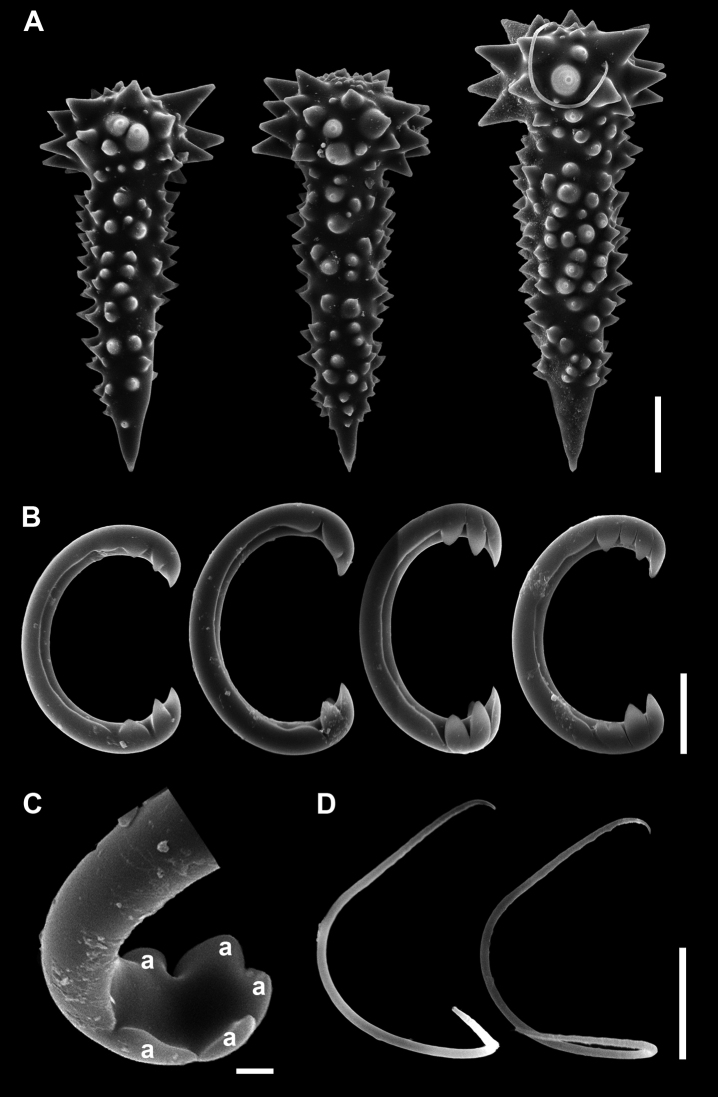
Spicules of *Discorhabdellahispida* sp. nov., holotype (NSMT-Po-2489). **A** Acanthostyles **B** isochelae **C** magnified view of one extremity of isochelae. a, alae **D** sigmoid microscleres. Scale bars: 20 µm (**A**); 10 µm (**B, D**); 2 µm (**C**).

Acanthostyles (Fig. 4A), club-shaped head with conical spines having blunt ends. Shaft straight, fusiform, and densely covered with prominent spines with tips sharply pointed, devoid of spines on the last 10–20 µm towards extremity. Terminal holes or orifices of spines especially around head could be detected. Size, 84.0–127.5 (103.6) µm in length, 41.1–57.7 (48.0) µm in head width including spines, 26.3–42.4 (31.1) µm in head width without spines, 24–35.9 (27.8) µm in width of shaft including spine, 16.2–27.5 (21.3) µm in width of shaft without spine.

Anchorate unguiferous isochelae (Fig. 4B, C), strongly curved C-shaped shaft with lateral expansion that forming a pair of fimbriae along its entire length. Both extremities bearing 3–7 short and unequal shaped alae. The alae closest to the lateral fimbriae sometimes reduced or nearly absent, and connected to the fimbriae. Size, 27.3–38.0 (31.7) µm in length, 2.9–4.0 (3.5) µm in shaft width.

Sigmoid microscleres (Fig. 4D), strongly curved shaft and irregular in shape. Size, 20.7–31.2 (26.3) µm in length, 0.7–1.0 (0.8) µm in shaft width.

##### Distribution.

Known only from type locality, Misaki, eastern part of Sagami Bay, Japan.

##### Etymology.

Specific epithet refers to its hispid surface appearance.

##### Remarks.

The present species appears well characterized by its spicule complement, especially its microscleres. The isochelae have a unique shape, with a strongly curved shaft compared to all other species of *Discorhabdella*, which have a straight or feebly curved shaft. However, the isochelae of *D.hispida* sp. nov. are similar to the anchorate isochelae of *Monanchoraunguiculata* (Dendy, 1922) (see also Lévi 1961, Vacelet et al. 1976). The presence of a sigmoid microsclere that is different from the true sigma, is also distinctive. Sigmas are present in four other *Discorhabdella* species: *D.hindei*; *D.littoralis*; *D.ruetzleri* and *D.urizae*; however, in these species, there are several differences in the other spicule characters (see Table 1).

*Discorhabdellahispida* sp. nov. differs from *D.hindei* by having acanthostyle (length: 84.0–127.5 µm) instead of pseudoastrose acanthostyles (length: 43–57 µm) in *D.hindei*, a less tuberculated base of the choanosomal styles and a less developed tyle of the ectosomal subtylostyles. It differs from *D.littoralis* by larger choanosomal subtylostyles (814–ca 1500 µm vs 117–300 µm), by having acanthostyles instead of pseudoastrose acanthostyles, and a more tuberculated base of choanosomal subtylostyles. It differs from *D.ruetzleri* by larger choanosomal subtylostyles (814–1500 µm vs 470–810 µm), larger acanthostyles (84.0–127.5 µm vs 17–40 µm), larger isochelae (27.3–38.0 µm vs 20–25 µm), absence of spined microxea. It differs from *D.urizae* by larger choanosomal subtylostyles (814–1500 µm vs 220–750 µm in length), absence of spined microxeas and a less tuberculated base of the choanosomal styles. Acanthostyles that are more than 90 µm long have been observed only in *D.tuberosocapitata* and in *D.misakiensis* sp. nov. described in this study. But both species lack sigmoid microscleres and have choanosomal subtylostyles with a well-developed lumpy base. Tubercles around the base of choanosomal subtylostyles are not well developed in *D.hispida* sp. nov. and can be comparable with those recently found in *D.pseudaster* and *D.ruetzleri*. However, *D.hispida* sp. nov. totally lacks peculiar pseudoaster of *D.pseudaster* and also lacks spined microxea of *D.ruetzleri*.

#### Discorhabdella misakiensis sp. nov.

Taxon classificationAnimaliaPoeciloscleridaCrambeidae

EEB6EE11-92D1-5A09-9704-DFCF290CD8C2

http://zoobank.org/636E3E9C-BF02-45D5-8CEE-222468D4C945

Figs 2D , 5 , 6

##### Material examined.

***Holotype*.**NSMT-Po-2490. Off Misaki, eastern part of Sagami Bay (Fig. 1), Japan (35°7.734'N, 139°34.133'E to 35°7.714'N, 139°34.061'E), 318–255 m depth, dredge, 10 January 2012.

##### Description of holotype.

***External morphology*.** Small, very thinly encrusting sponge, about 0.2 mm thick, with velvet surface, white in alcohol. Size, 8 × 5 mm (Fig. 2D). Ostia and oscules not observed either in live or in the preserved specimen.

***Skeleton*.** Hymedesmioid skeleton made by choanosomal subtylostyles and acanthostyles. Choanosomal subtylostyles mostly arranged perpendicular to surface with tips oriented upward. Anchorate unguiferous isochelae distributed in whole body.

**Figure 5.**
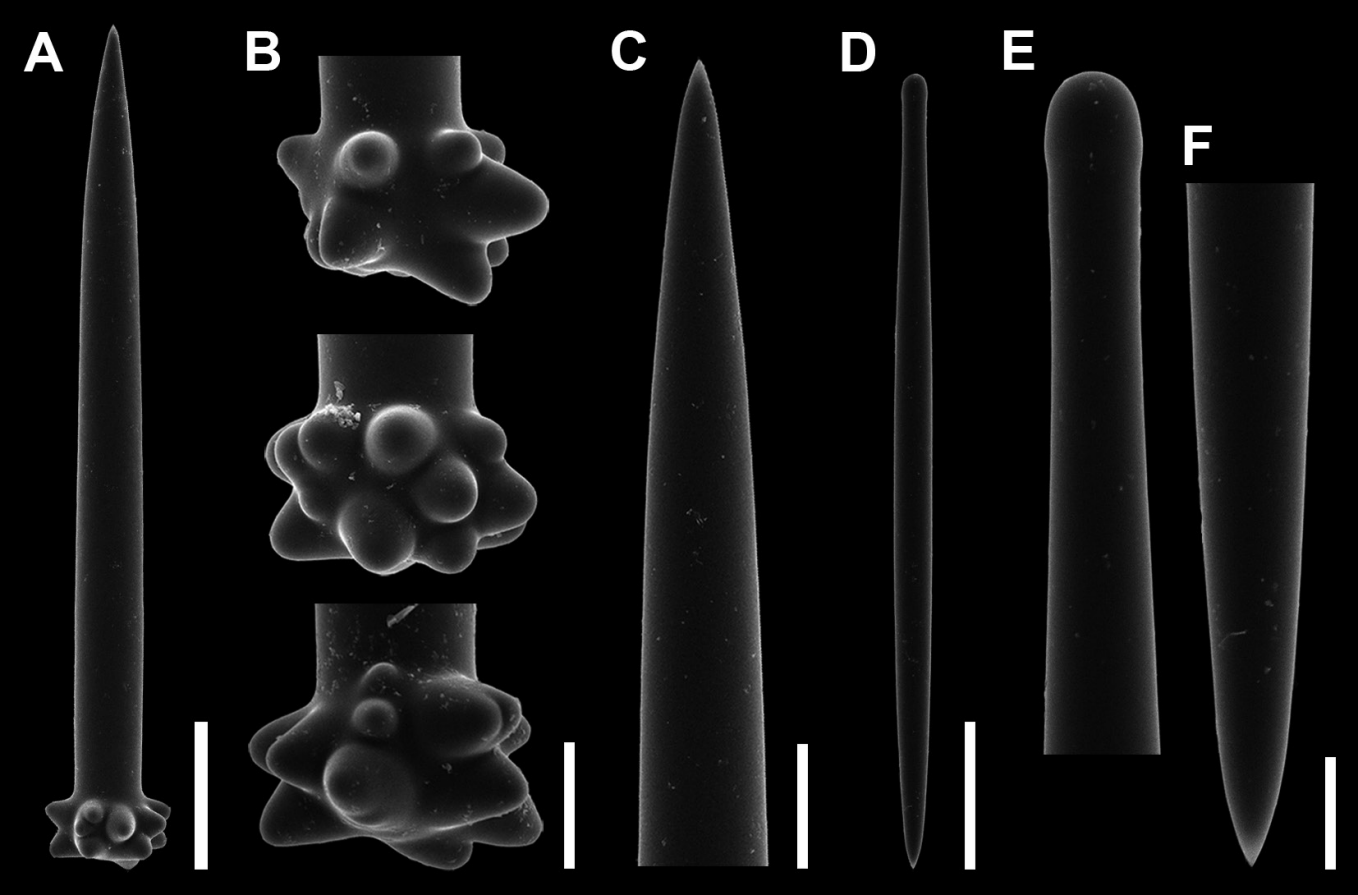
Spicules of *Discorhabdellamisakiensis* sp. nov., holotype (NSMT-Po-2490). **A–C** Choanosomal subtylostyle **B** magnified view of base of subtylostyle with prominent lumpy projections **C** tip **D–F** ectosomal subtylostyle **E** tyle **F** tip. Scale bars: 50 µm (**A, D**); 20 µm (**B, C**); 10 µm (**E, F**).

***Spicules*.** Choanosomal subtylostyles (Fig. 5A–C), straight, almost uniform in thickness along shaft gradually tapering to a sharp point (Fig. 5C). Lumpy base bearing many prominent smooth projections (Fig. 5B). Size, 252– 336.4 (295.2) µm in length, 18.6– 26.6 (22.6) µm in shaft width, 33.2–45.6 (40.2) µm in base width.

Ectosomal subtylostyles (Fig. 5D–F), fusiform, smooth and straight, with smooth and slightly swollen tyle (Fig. 5E). Maximum diameter at middle region, then gradually tapering to sharp point (Fig. 5F). Size, 203–257 (232) µm in length, 10.6–14.1 (11.7) µm in shaft width, 7.9–9.9 (8.9) µm in tyle width.

**Figure 6.**
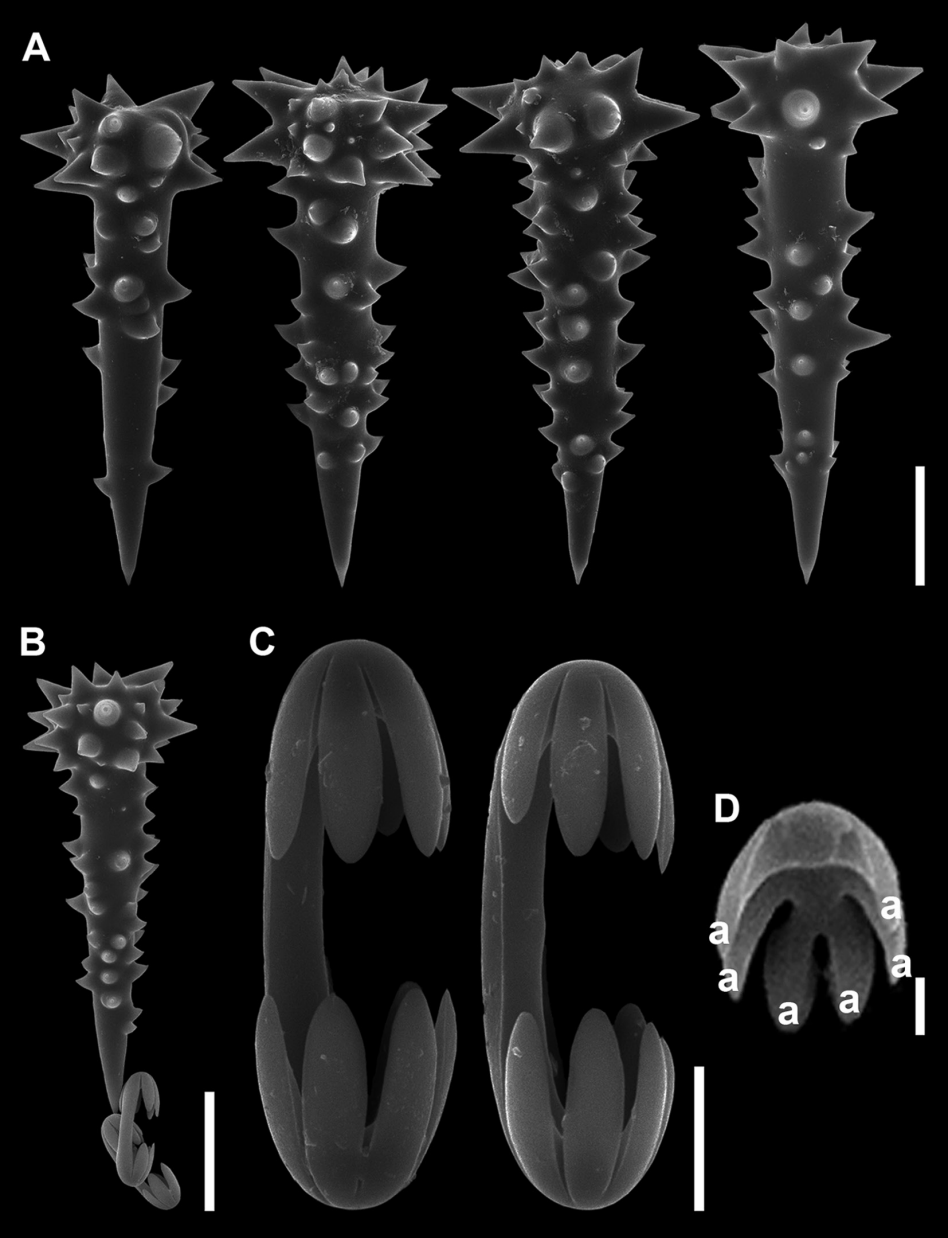
Spicules of *Discorhabdellamisakiensis* sp. nov., holotype (NSMT-Po-2490). **A** Acanthostyles **B** comaparative view of acanthostyle and isochelae **C** isochelae **D** back side view of broken isochelae. a, alae. Scale bars: 20 µm (**A, B**); 5 µm (**C**); 2 µm (**D**).

Acanthostyles (Fig. 6A, B), straight, surface covered with prominent spines especially at club-shaped head with longer spines. Spines on shaft slightly recurved with tips sharply pointed. Shaft devoid of spines from extremity up to ca. 10–20 µm. Size, 73–91.3 (82.0) µm in length, 27.9–42.0 (34.2) µm in head width including spines, 15.6–21.8 (19.8) µm in head width without spines.

Anchorate unguiferous isochelae (Fig. 6B–D), shaft nearly straight, with a pair of fimbriae along whole shaft; bearing 6 alae (Fig. 6B–D). Size, 17.5–21.9 (19.8) µm in total length, 2.0–2.7 (2.2) µm in shaft width, 6.7–8.0 (7.3) µm in alae length.

##### Distribution.

Known only from type locality, Misaki, eastern part of Sagami Bay, Japan.

##### Etymology.

Specific epithet refers to type locality: Misaki.

##### Remarks.

*Discorhabdellamisakiensis* sp. nov. has only isochelae as microscleres. This composition of spicules can be found in one other species of the genus, *D.tuberosocapitata* from Azores, Canaries and Madeira (Van Soest 2002, Van Soest et al. 2019). The two species can be clearly differentiated by the size of their spicules: all spicules are smaller in *D.misakiensis* sp. nov. (see Table 1). In addition, they can be differentiated by the shape of their isochelae. Although the isochelae of *D.tuberosocapitata* and *D.misakiensis* sp. nov. have similar number of alae, the alae in *D.tuberosocapitata* are more widely opened. The reported number of isochelae alae in *D.tuberosocapitata* is rather confusing because different authors reported different number of alae despite all of them observing the same type material: four in Boury-Esnault et al. (1992), four to five in Van Soest (2002) and seven to eight in Maldonado and Uriz (1996). This is possibly due to differences in the interpretation of the fused alae. Boury-Esnault et al. (1992) and Van Soest (2002) considered the two alae fused at the base as one, while Maldonado and Uriz (1996) counted them as two. The alae number of *D.misakiensis* sp. nov. is here counted as six; however, the two frontal alae seem to fuse at the base or might be regarded as one ala divided into two (Fig. 6D). Further evidence of separation of these two species is their distant geographical distribution: *D.tuberosocapitata* is reported from Azores, Canaries and Madeira (Van Soest 2002, Van Soest et al. 2019) but *D.misakiensis* sp. nov. is found only from the type locality, Sagami Bay, Japan. The dichotomous central ala is also found from “eight-toothed isochelae” of *D.hindei* (Maldonado and Uriz 1996); however, *D.misakiensis* sp. nov. and *D.hindei* are clearly separated by the possession of sigma in the latter species. Furthermore, *D.hindei* has been reported only from Alboran Sea (Maldonado and Uriz 1996), which is very distant from type locality of *D.misakiensis* sp. nov.

The choanosomal subtylostyles of the new species are relatively small, and their length overlapped with that of the ectosomal subtylostyles. In *Discorhabdella*, this pattern is found only in *D.littoralis* (see Table 1). However, *D.littoralis* and *D.misakiensis* sp. nov. are clearly separated by the size of acanthostyles (26–40 µm vs 73.0–91.3 in length), the presence of isochelae (absent in *D.littoralis*), and of sigmas (absent in *D.misakiensis* sp. nov.). *D.littoralis* has been only reported from off the Pacific coast of Panama (Maldonado et al. 2001), which also exhibits distant geographical distribution from type locality of *D.misakiensis* sp. nov.

##### Discussion.

The present study adds two new species to the genus *Discorhabdella*, which now has nine species. This is the first record of the genus and family Crambeidae from Japanese waters. Thus the discovery of these two new species from warm temperate northwest Pacific extends the geographical distribution of the genus (see Table 1).

Vacelet and Cárdenas (2018) raised doubts to the hypothetical polyaxial nature of the choanosomal styles/subtylostyles and the pseudoastrose acanthostyles that has been proposed by Uriz and Maldonado (1995) and Maldonado and Uriz (1996). The authors proposed instead, a monaxonal origin for the spicule shaft with secondary axes for bulges. In our study, we could not precisely distinguish axes on the choanosomal subtylotyles or the acanthostyles.

Feeble microspines around the distal tips of ectosomal subtylostyles have been first reported from *Crambetuberosa* Maldonado & Benito, 1991 and later considered as a possible common character of the genera *Discorhabdella* and *Crambe*, both in the family Crambeidae (Maldonado and Uriz 1996). In this study, this character was observed in *D.hispida* sp. nov. (e.g. Fig. 3F) but seems to be absent in *D.misakiensis* sp. nov. (Fig. 5F). This character was not mentioned in the recently described species, *D.pseudaster* and *D.ruetzleri* (Vacelet and Cárdenas 2018, Díaz and Pomponi 2018). The actual affinity between *Discorhabdella* and *Crambe* has not been revealed as yet (Maldonado and Uriz 1996), but the feeble microspines around the distal tips of the ectosomal subtylostyles may be a symplesiomorphy for these two genera.

The evolutionary aspect of morphological divergence among sphaeroclones, pseudoastrose acanthostyles, and typical acanthostyles has long been discussed and the question remains as to whether the amount of change between sphaeroclones and astrose acanthostyles is more important than the whole set of shared morphological features in determining the phylogenetic relationships between *Crambe* and *Discorhabdella* (Uriz and Maldonado 1995, Maldonado and Uriz 1996). Our findings on the two new species add more knowledge on acanthostylose derivatives in *Discorhabdella*. To date, long acanthostyles have been found only in *D.tuberosocapitata* (with ca 130 µm in length), but in all other species of *Discorhabdella* they are less than 60 µm (see Table 1) and thus regarded as pseudoastrose acanthostyle because of the putative polyaxial nature contrasting the monoaxial nature of typical acanthostyles of other demosponge taxa (Uriz and Maldonado 1995, Maldonado and Uriz 1996). In the two new species, acanthostyles are longer than 70 µm in length, which means the alleged possession of long acanthostyles differing from typical pseudoastrose acanthostyles, is not unusual in *Discorhabdella*. They also provide clues for solving the trait of gradual morphological divergence between sphaeroclones, pseudoastrose acanthostyles, and acanthostyles along with pseudoaster recently found from *D.pseudaster* (Vacelet and Cárdenas 2018). A molecular phylogenetic study is necessary to unravel the diversification of sphaeroclones, pseudoastrose acanthostyles, acanthostyles and pseudoasters as well as the affinity of *Discorhabdella* and *Crambe* within the order Poecilosclerida (Maldonado and Uriz 1996, Vacelet and Cárdenas 2018).

### Identification key to species of extant *Discorhabdella*

**Table:** 

1	Pseudoasters present	***D.pseudaster***
–	Pseudoasters absent	**2**
2	Chelae present	**3**
–	Chelae absent	***D.littoralis***
3	Microscleres isochelae only	**4**
–	More types of microscleres in addition to isochelae	**5**
4	Size of choanosomal subtylostyles much larger than those of ectosomal subtylostyles	***D.tuberosocapitata***
–	Size of choanosomal subtylostyles overlapping with those of ectosomal subtylostyle	***D.misakiensis* sp. nov.**
5	Standard sigmas present	**6**
–	Standard sigmas absent	**7**
6	Spinose microxea present	**8**
–	Spinose microxea absent	***D.hindei***
7	Other sigmoid microscleres present	***D.hispida* sp. nov.**
–	Other sigmoid microscleres absent	***D.incrustans***
8	Ectosomal subtylostyles longer than 250 µm	***D.ruetzleri***
–	Ectosomal subtylostyles shorter than 250 µm	***D.urizae***

## Supplementary Material

XML Treatment for Discorhabdella

XML Treatment for Discorhabdella hispida

XML Treatment for Discorhabdella misakiensis
